# Gait analysis using a suit-type wearable device in rehabilitation therapy after stroke: A case report

**DOI:** 10.1097/MD.0000000000043137

**Published:** 2025-07-04

**Authors:** Kimito Mio, Takatsugu Okamoto, Yasuhide Nakayama, Daigo Sakamoto, Kentaro Yoshida, Masahiro Abo

**Affiliations:** a Department of Rehabilitation Medicine, Jikei University School of Medicine, Tokyo, Japan; b Department of Rehabilitation, Nishi Hiroshima Rehabilitation Hospital, Hiroshima, Japan.

**Keywords:** gait analysis, rehabilitation therapy, suit-type wearable device

## Abstract

**Rationale:**

In poststroke patients with hemiplegia, gait disorders often remain as a sequela. Although gait analysis commonly focuses on lower limb dysfunction, there is limited evidence addressing the contribution of upper limb movement to gait improvement after stroke. A 3D optical motion analysis is commonly used to treat gait disorders, as it allows precise assessment of gait disorders by measuring joint range of motion, stride length, and time. However, these require multiple cameras and sensors, making the setup time-consuming. In this case, we conducted gait analysis using a newly introduced and easier-to-use suit-type wearable device (STWD) before and after rehabilitation therapy in a chronic posthemorrhagic stroke patient.

**Patient concerns:**

An 82-year-old Japanese man with right hemiplegia and gait disorder due to left midbrain hemorrhage was referred to our hospital for rehabilitation therapy.

**Diagnoses:**

By using STWD, we determined that his gait disorder was mainly due to right upper limb hemiplegia, as STWD showed that the right shoulder flexion angle during gait was very limited.

**Interventions:**

We performed botulinum toxin type A therapy and repetitive transcranial magnetic stimulation for the upper limb, as well as physical and occupational therapy for upper limb and gait disorders.

**Outcomes:**

Right shoulder flexion angle during gait, upper limb function assessed by Fugl-Meyer Assessment, and gait function assessed by Timed Up and Go test all improved. Each STWD session took about 2 to 3 minutes to set up.

**Lessons:**

When treating gait disorders, we should not only look at the patients’ lower limb function but also their upper limb function as well. STWD is more convenient than the conventional 3-dimensional optical motion analysis, allowing for quick and less burdensome measurements. Its application in various scenarios is anticipated.

## 1. Introduction

Patients often experience hemiparalysis after a stroke, leading to gait disorders. Gait disorders are generally evaluated by gait speed and patterns. For more detailed evaluation, a 3D optical motion analysis is often used. This allows for an objective assessment of gait patterns but requires extensive preparation and is complex to operate.

While many reports emphasize the effectiveness of approaches targeting lower limb motor paralysis, there are also reports suggesting that addressing upper limb motor paralysis can improve gait function as well.^[1]^ However, limited prior research has examined this relationship using wearable motion analysis devices.

We herein report a case which upper limb rehabilitation treatment improved gait disorders. Using a new motion analysis device, a suit-type wearable device (STWD), we were able to assess the improvement in gait patterns more conveniently compared to the conventional 3D optical motion analysis.

## 2. Case

**Figure 1.**
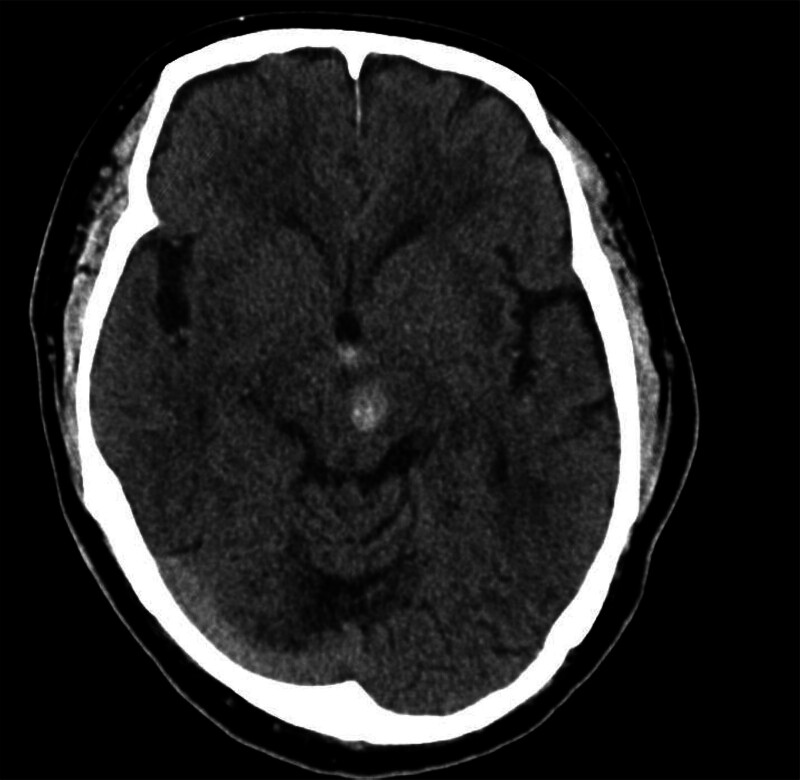
CT image of midbrain hemorrhage.

The patient was a 60-year-old man who experienced a left midbrain hemorrhage 2 years ago (Fig. 1) and received conservative treatment. After discharge, he continued physical therapy at home for twice a week, which focused on gait disorder though the effect was limited. He visited our hospital to consult about further rehabilitation treatment plans. His past medical history included hypertension, deep vein thrombosis, pulmonary embolism and hepatitis C. There was nothing to note in his family history or allergy.

The physical examination at the initial visit was as follows: clear consciousness and able to converse, with no apparent higher brain dysfunction. Right hemiplegia was seen, with the upper limb Brunnstrom Recovery Stage (BRS) at Ⅳ, fingers at Ⅴ, and lower limb at Ⅲ, indicating moderate paralysis. For upper limb function, Fugl-Meyer Assessment (FMA) was at 26 points, and the modified Ashworth scale (MAS) was 2 for shoulder extension, 2 for elbow extension, and 1 + for wrist dorsiflexion, indicating moderate upper limb impairment and spasticity. Joint range of motion showed restrictions in right shoulder flexion at 100 degrees, right forearm pronation at 30 degrees, and right wrist dorsiflexion at 0 degrees. The right shoulder joint was constantly in about 10 degrees of extension. Sensory loss or abnormalities were not observed. The patient was able to walk indoors and outdoors with a cane but he did not use orthoses. While walking outdoors, he sometimes received family assistance as he visited our hospital with his wife. A head CT scan upon admission at our hospital showed no new hemorrhage or infraction, and the hematoma from midbrain hemorrhage was completely absorbed. Blood tests revealed no notable abnormities.

Based on the medical history and physical findings, a rehabilitation treatment combining botulinum toxin type A (BoNT-A) therapy and repetitive transcranial magnetic stimulation (rTMS) was planned to improve gait disorders and upper limb function. 28 days after the initial visit (y + 28), BoNT-A therapy was performed on an outpatient basis, with 350 total units of botulinum toxin type A (BOTOX®) injected into the following muscles; latissimus dorsi (100 units), subscapularis (50 units), biceps brachii (50 units), triceps brachii (50 units), pronator teres (50 units), and flexor carpi radialis (50 units). rTMS combined rehabilitation treatment on inpatient basis was started on day 77 (y + 75) and continued for 13 days until day 85 (y + 88). rTMS was applied to the right upper limb motor area with low-frequency stimulation (1 Hz) at 2400 pulses per day (100% of resting motor threshold). Physical therapy and occupational therapy were each conducted for 60 min per day. Physical therapy focused mainly on gait, emphasizing on right upper limb arm swing practice. Occupational therapy included range of motion exercises, stretching, and upper limb functional training. During the period between BoNT-A therapy and impatient rTMS combined rehabilitation therapy, the patient continued rehabilitation therapy at home.

**Figure 2.**
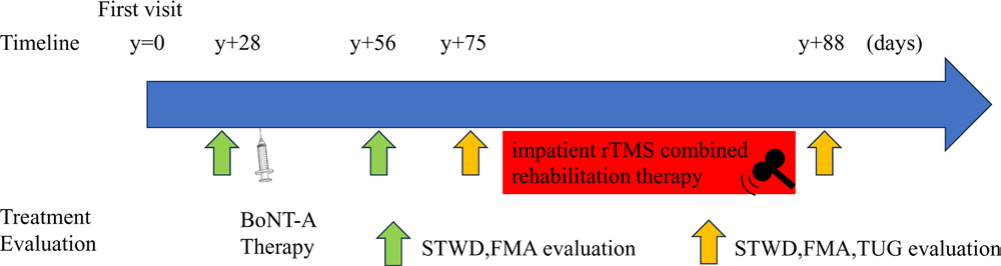
Clinical course of the present case. BoNT-A = botulinum toxin type A, FMA = Fugl-Meyer assessment, STWD = suit-type wearable device, TUG = timed up and go test.

The evaluation was conducted as follows (Fig. 2). Gait pattern evaluation using STWD and upper limb function evaluation using FMA were performed before BoNT-A therapy (y + 28) and 4 weeks after BoNT-A therapy (y + 56). Additionally, gait function was evaluated using Timed Up and Go (TUG) test before (y + 75) and after (y + 88) the rTMS combined rehabilitation therapy.

**Figure 3.**
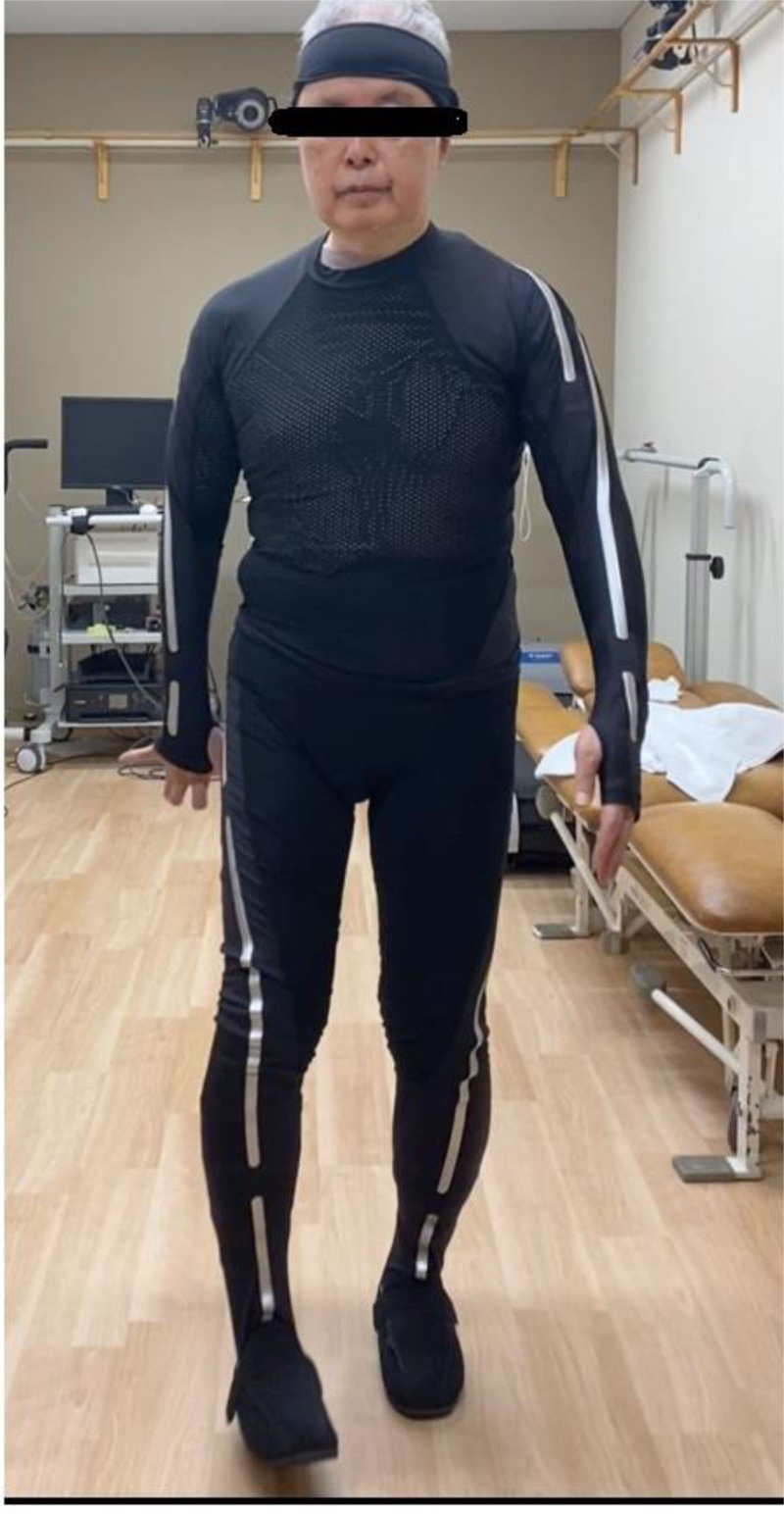
Image of STWD. STWD = suit-type wearable device.

The STWD which we used was Xenoma’s e-skin MEVA®. (Fig. 3) The suit-type device was easy to wear and did not require additional sensors. 18 sensors are embedded to the bodysuit and wirelessly transmitted data to a computer, allowing evaluation with ease. The sampling frequency was 100 Hz, enabling detailed data acquisition. Previous studies have explored Xenoma’s e-skin MEVA® applicability for motion analysis. For instance, Oyama et al demonstrated its reliability and validity in quantifying lower limb kinematics during walking in healthy adults, showing promise for broader clinical applications in gait analysis and rehabilitation contexts.^[2]^

We focused on the right shoulder joint flexion angle and the arm swing amplitude during gait. The right shoulder joint flexion angle was calculated as the average of right shoulder flexion angles during the 2nd to the 5th step after the start of the gait analysis, and we defined the flexion angles as positive values and extension as negative values. Arm swing amplitude was analyzed as the absolute value of the difference between the maximum flexion angle and extension angle of the right shoulder during the 2nd to the 5th step after the start of the gait analysis. Although trunk sway was observed during gait analysis, shoulder flexion-extension angles were calculated based on sensors placed on the lateral sides of the trunk, minimizing the impact on measurements.

**Figure 4.**
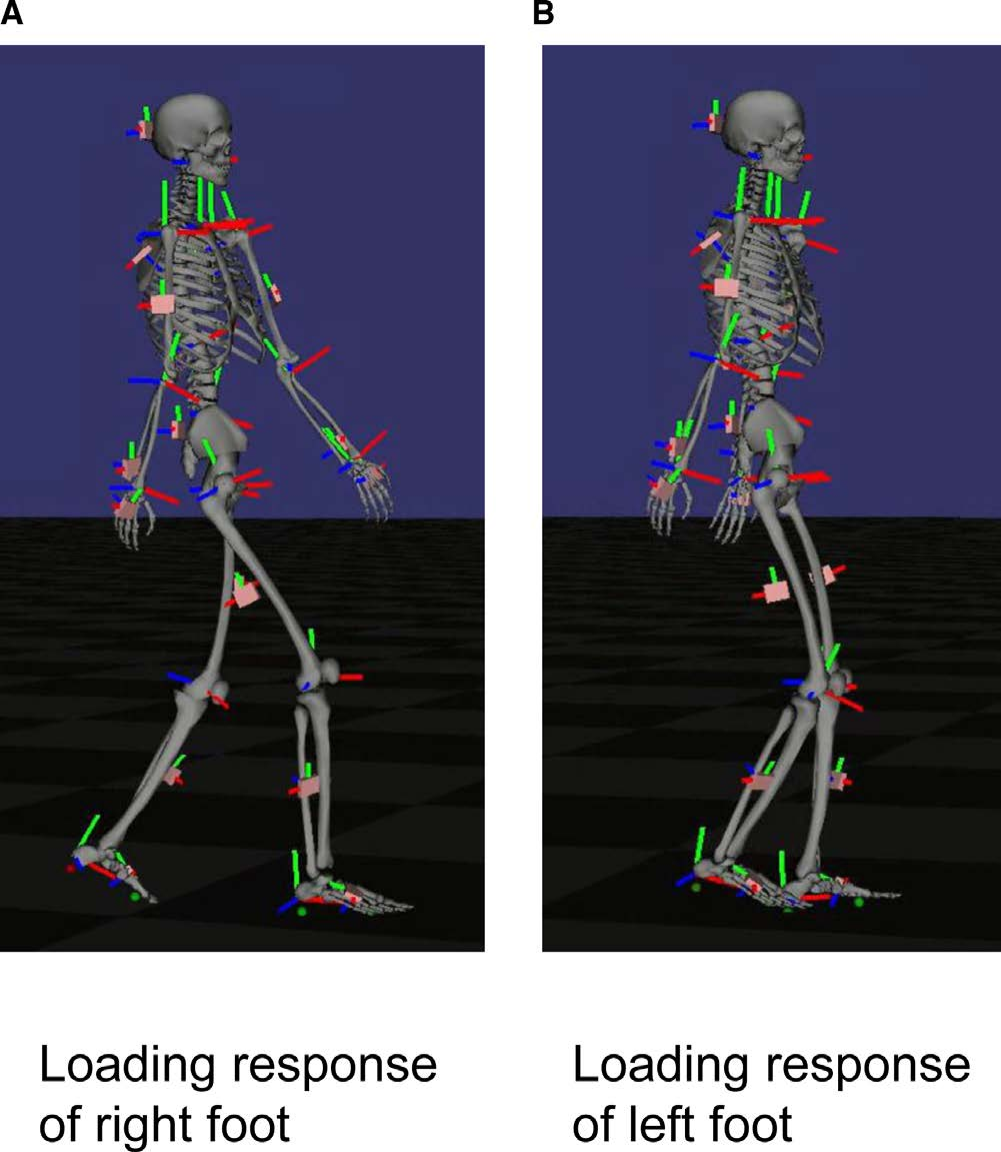
Result of gait analysis using STWD before BoNT-A therapy. (A) Loading response of right foot. (B) Loading response of left foot. BoNT-A = botulinum toxin type A, STWD = suit-type wearable device.

**Figure 5.**
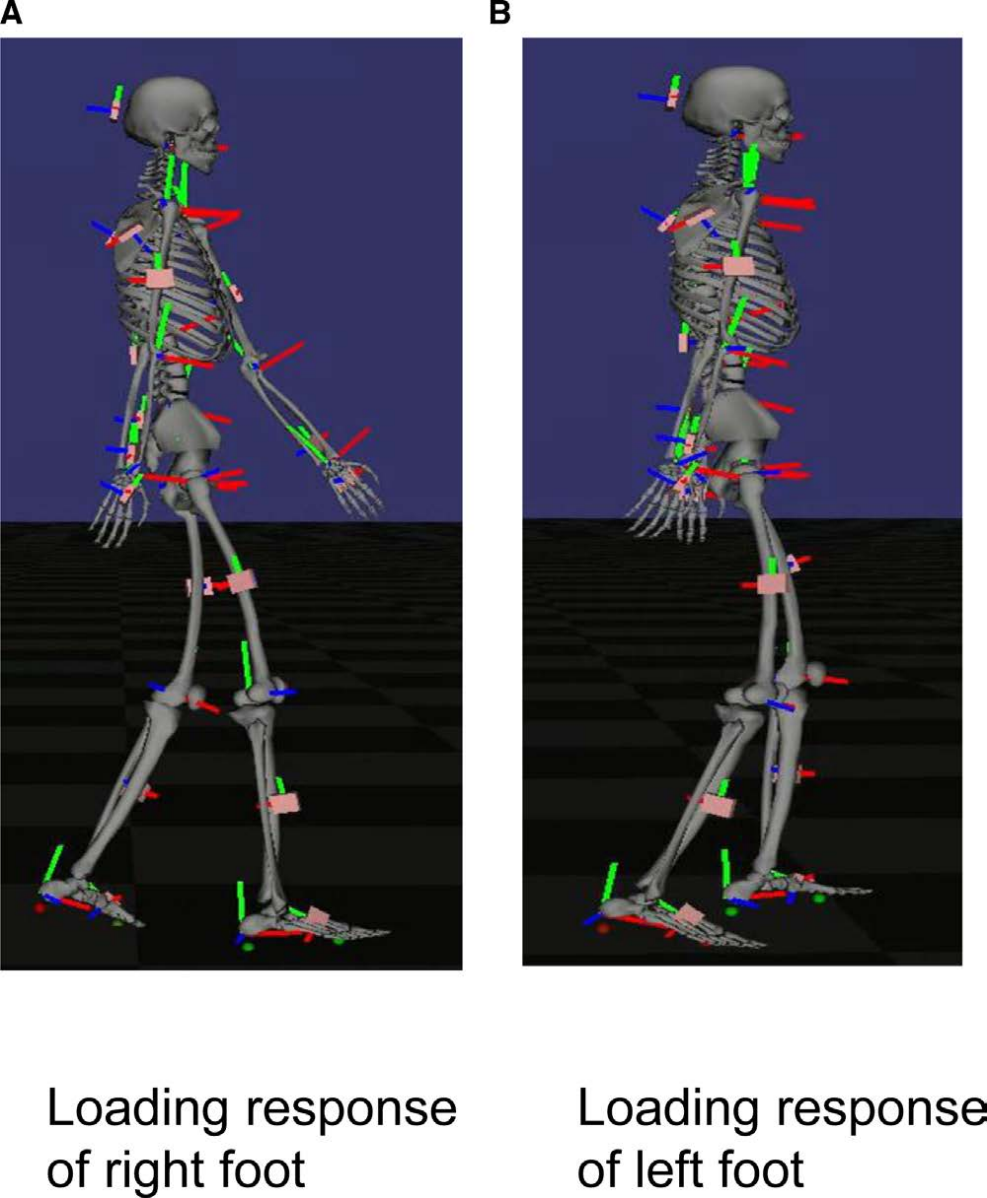
Result of gait analysis using STWD 4 wk after BoNT-A therapy. (A) Loading response of right foot. (B) Loading response of left foot. BoNT-A = botulinum toxin type A, STWD = suit-type wearable device.

**Figure 6.**
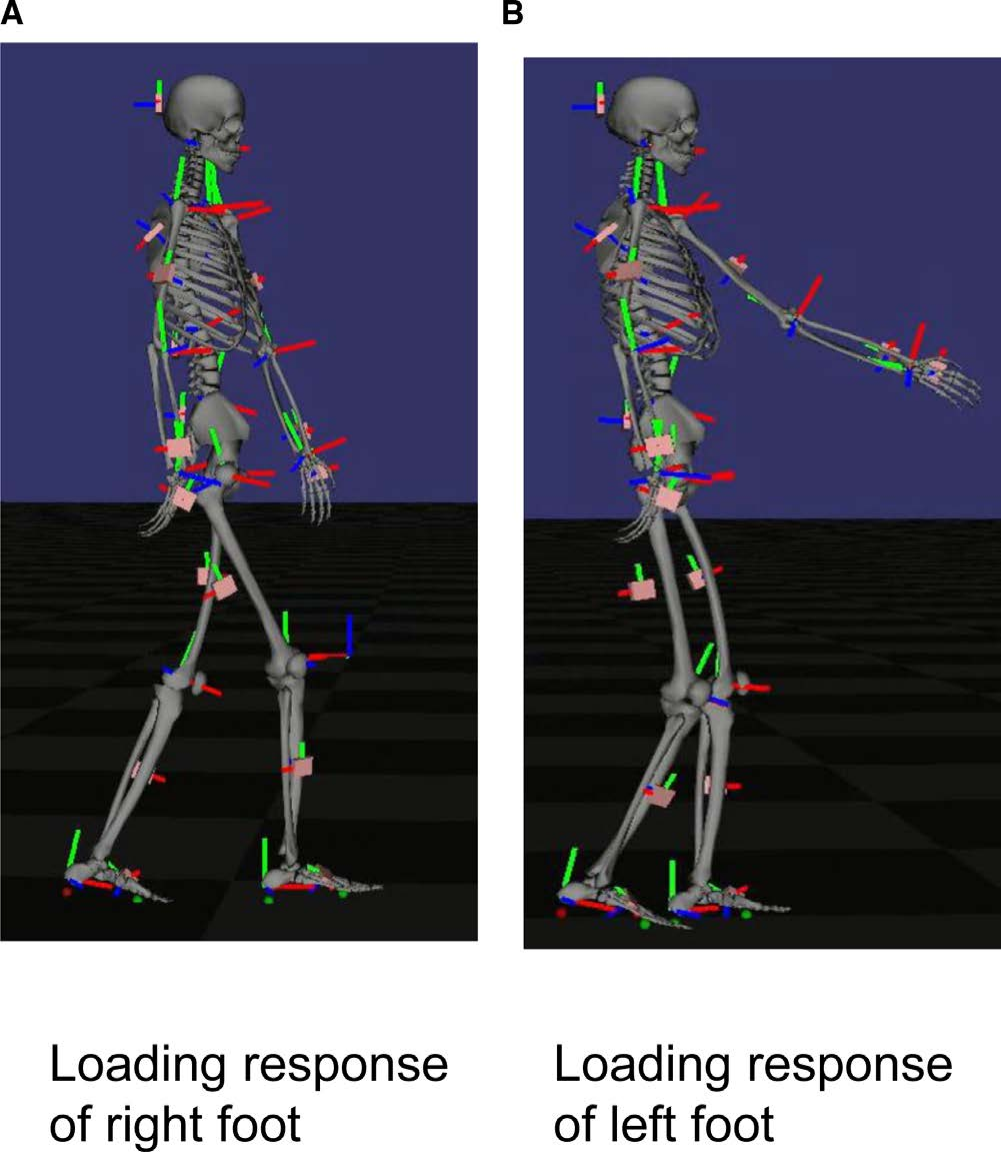
Result of gait analysis using STWD before rTMS combined rehabilitation therapy. (A) Loading response of right foot. (B) Loading response of left foot. STWD = suit-type wearable device, rTMS = transcranial magnetic stimulation.

**Figure 7.**
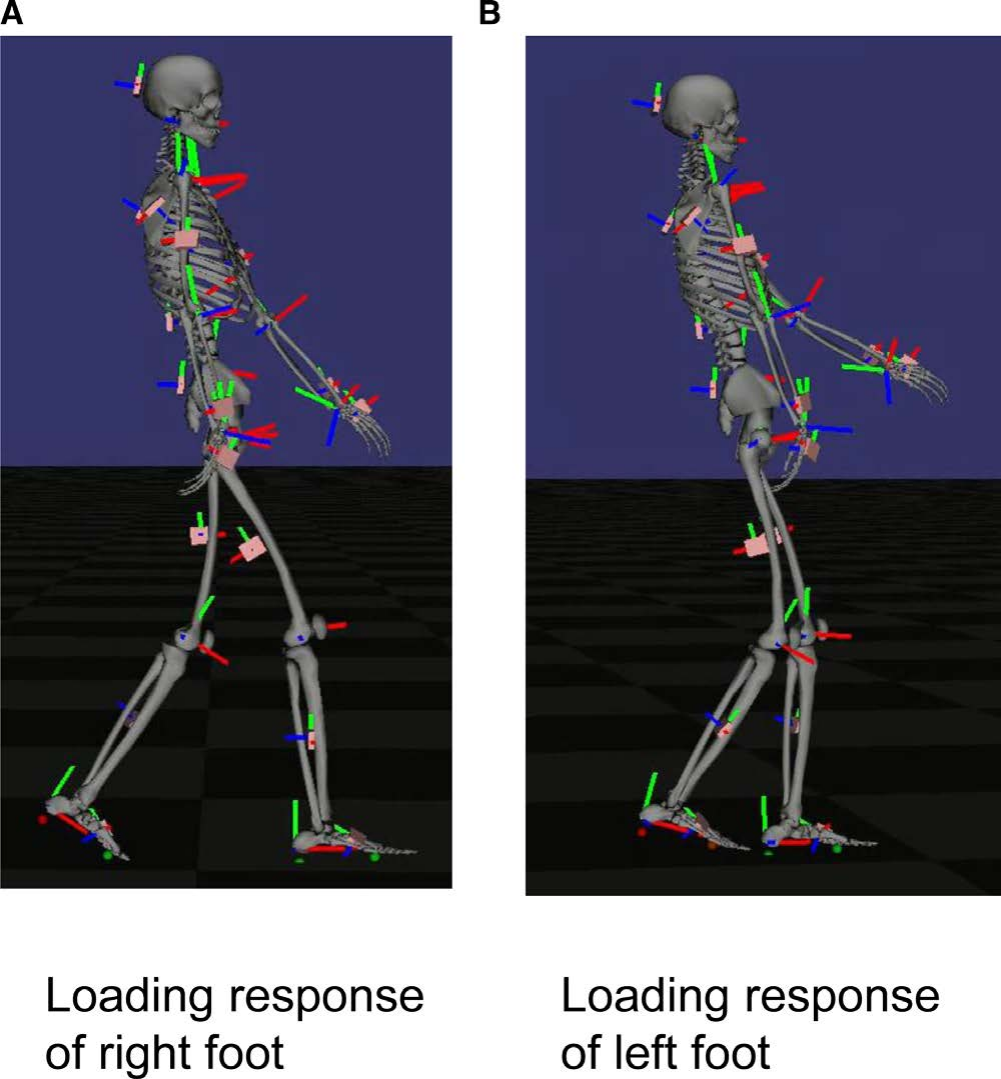
Result of gait analysis using STWD after rTMS combined rehabilitation therapy. (A) Loading response of right foot. (B) Loading response of left foot. STWD = suit-type wearable device, rTMS = transcranial magnetic stimulation.

**Figure 8.**
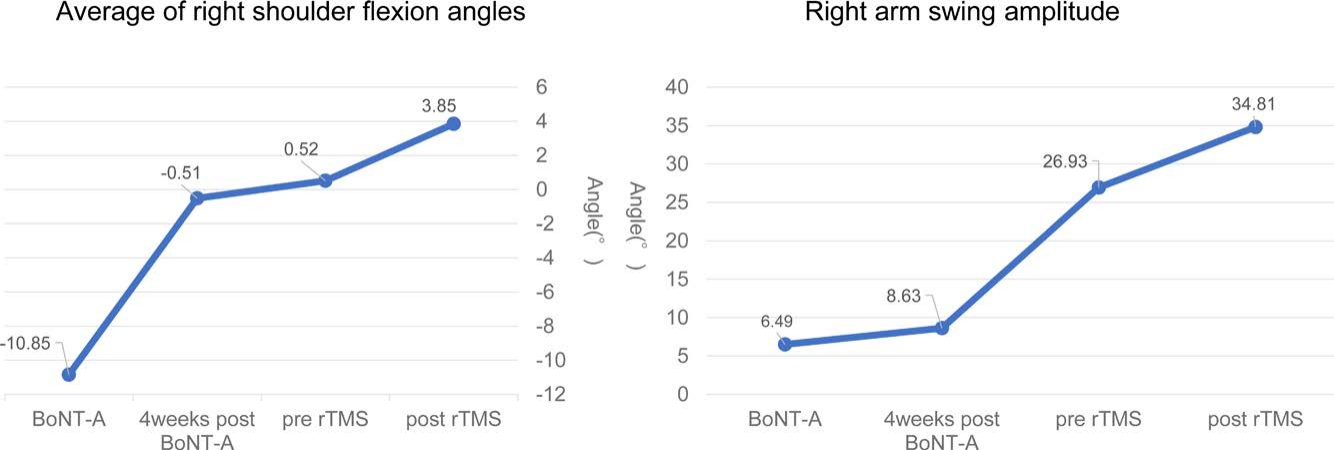
Graph of average right shoulder flexion angles and right arm swing amplitude.

**Figure 9.**
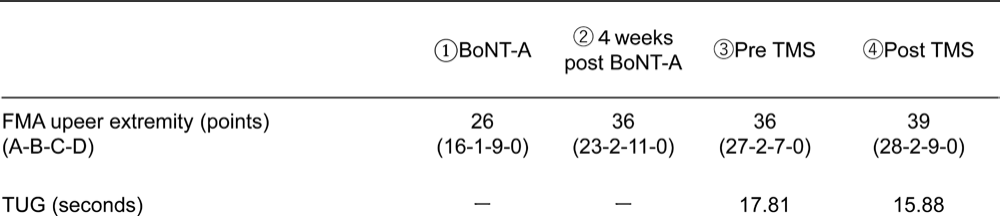
Scores of FMA and TUG. FMA = Fugl-Meyer assessment, TUG = timed up and go test.

The STWD measurement results visually confirmed the improvement in the right shoulder joint flexion angle during gait. (Fig. 4: before BoNT-A therapy, initial evaluation; Fig. 5: 4 weeks after BoNT-A therapy; Fig. 6: before rTMS combined rehabilitation treatment; Fig. 7: after rTMS combined rehabilitation treatment, final evaluation) the average right shoulder flexion angle and arm swing amplitude (Fig. 8) improved from −10.85° at the initial evaluation to 3.85° at the final evaluation, and the arm swing amplitude improved from 6.49° to 34.81°, respectively. The FMA upper limb score (Fig. 9) also improved over time, notably 4 weeks after BoNT-A therapy. The TUG (Fig. 9) improved from 17.81 second to 15.88 seconds. STWD was applicable to this case, with preparation for measurement, including computer startup, patient STWD attachment, and calibration of STWD measurement markers in three postures, was completed in about 2–3 min overall. There were no complaints of discomfort from the patient during the test.

## 3. Discussion

This case involved a patient with moderate right hemiparesis due to left midbrain damage, where the right shoulder was constantly in an extension position. Intensive upper limb therapy combined with BoNT-A therapy and rTMS combined rehabilitation therapy led to improvement in upper limb function, resulting in improved arm swing and gait ability.

In patients with post-stroke upper limb dysfunction, BoNT-A therapy has been reported to improve upper limb paralysis, spasticity, and range of motion.^[3,4]^ rTMS has also been reported to improve FMA upper limb scores, paralysis, and spasticity.^[5]^ These combination therapies are considered most effective when combined with intensive rehabilitation.^[6]^ Conversely, many reports have highlighted improvements in gait patterns and speed when BoNT-A therapy or rTMS targets the lower limbs,^[7,8]^ but reports on improvements in gait patterns and speed when targeting the upper limbs are scarce.

Furthermore, focusing on shoulder flexion angle and gait improvement is unprecedented, demonstrating the novelty of this case report.

In this case, significant improvements in the FMA upper limb score and shoulder flexion angle were observed after the BoNT-A therapy. This improvement in shoulder range of motion and FMA is considered due to reduced spasticity around the shoulder joint and improved upper limb function from BoNT-A therapy. Although no significant changes in arm swing amplitude were observed 4 weeks after BoNT-A therapy, this could be attributed to the fact that rehabilitation therapy was only done twice a week a home. However, notable improvements in arm swing amplitude were observed at the start of rTMS, indicating the combined effect of BoNT-A therapy and the accumulation of rehabilitation training.

Two weeks of impatient rTMS combined rehabilitation therapy focusing on gait and upper limb functional resulted in further improvements in all parameters (upper limb function, gait ability, shoulder flexion angle, and arm swing amplitude). Notably, the improvement in shoulder flexion range of motion likely contributed to the enhanced TUG performance. Previous reports have shown that gait with arms crossed or with fixed shoulders significantly reduces speed and stride compared to normal arm swing gait.^[9]^ In this case, pretreatment gait consistently involved right shoulder extension. After BoNT-A therapy and impatient rTMS combined rehabilitation therapy, shoulder joint flexion angle improved, leading to a more normal arm swing during gait and significant TUG improvement.

In this study, the use of STWD allowed us to observe upper limb movements during gait, which are difficult to analyze with conventional evaluation methods. While traditional 3D motion analysis is highly regarded for their accuracy, they have several reported disadvantages, including high cost, complexity of operation and long evaluation times, the need for numerous cameras and other equipment requiring significant space, and the necessity of attaching multiple markers to the patient, which can hinder their natural movements.^[10]^ The STWD used in this study overcame all these disadvantages except for cost. It was found to be very useful in this case, with no complaints from the patient. However, one of the limitations of this report is that we did not compare the accuracy of STWD with that of conventional 3D motion analysis systems. Future studies are warranted to validate its precision in clinical populations and assess its consistency.

## Author contributions

**Conceptualization:** Kimito Mio.

**Writing – original draft:** Kimito Mio, Takatsugu Okamoto, Yasuhide Nakayama, Daigo Sakamoto, Kentaro Yoshida.

**Writing – review & editing:** Kimito Mio, Takatsugu Okamoto, Yasuhide Nakayama, Daigo Sakamoto, Kentaro Yoshida.

**Supervision:** Masahiro Abo.
